# Paraquat Poisoning: Insights from Autopsy, Histology, and Liquid Chromatography with Tandem Mass Spectrometry in Multidisciplinary Forensic Toxicology Practice

**DOI:** 10.3390/toxics12090675

**Published:** 2024-09-16

**Authors:** Issarapong Nuwongsa, Tanyarat Markmee, Nareerat Pholpo, Manoch Chockjamsai, Tawachai Monum, Yutti Amornlertwatana, Preechaya Tajai

**Affiliations:** Department of Forensic Medicine, Faculty of Medicine, Chiang Mai University, Chiang Mai 50200, Thailand; issarapong.nu@cmu.ac.th (I.N.); tanyarat.markmee@cmu.ac.th (T.M.); nareerat.p@cmu.ac.th (N.P.); manoch.c@cmu.ac.th (M.C.); tawachai.m@cmu.ac.th (T.M.); yutti.amornlert@cmu.ac.th (Y.A.)

**Keywords:** paraquat, poisoning, autopsy, histology, LC-MS/MS

## Abstract

The herbicide paraquat (PQ) is responsible for a significant number of fatalities resulting from self-poisoning. Nevertheless, only a limited number of comprehensive studies focusing on fatal PQ poisoning, which include examination of autopsy findings, histopathology, and quantitative analysis of post-mortem samples, have been published. This study aimed to evaluate autopsy findings, histopathology, and quantitative analysis of PQ in post-mortem human serum samples using liquid chromatography-tandem mass spectrometry (LC-MS/MS), a simple, sensitive, and specific method. Autopsies were performed on all deaths due to PQ poisoning, and serum samples were sent to the toxicology laboratory for chemical analysis. The method was successfully applied to seven human serum samples, and the results indicate its reliability for detecting PQ. The study reports fatal serum PQ levels ranging from 0.5 to 372.0 µg/mL. The comprehensive data presented in this study can be useful for further research and practical applications.

## 1. Introduction

Acute poisoning, caused by poison exposure over a short period of time, is a serious public health concern worldwide [1]. The reported poisoning cases in many countries in Asia, such as Taiwan, India, and Sri Lanka, are intentional and often involve pesticides [2,3]. In Thailand, almost all lethal cases were attributed to pesticide poisoning [3]. Although paraquat (PQ) has been banned in over 67 countries, it continues to be widely used in several others, particularly across Asia and Latin America [4]. As such, PQ remains one of the most lethal pesticides and continues to be a significant public health concern [4,5].

Paraquat (1,1′-dimethyl, 4,4′-bipyridinium dichloride; PQ) is an effective herbicide that can cause potential health hazards with a high mortality rate in acute poisoning [6,7,8]. Self-poisoning with PQ ingestion is a major cause of morbidity and mortality in the Asia-Pacific region [9]. In Thailand, pesticides were responsible for 41.5% of cases involving acute poisoning. Insecticides comprised 50% of pesticide-related cases, with herbicides following at 24.7%, where glyphosate and PQ were the primary herbicides implicated [3]. Acute PQ poisoning in Thailand has received limited attention in research. Previous studies have indicated survival periods ranging from 26 h to 59 days [10], along with a high mortality rate of 71% [11]. Intracellular toxicity of PQ is believed to stem from its ability to generate reactive oxygen species (ROS) through redox cycling and disrupt the mitochondrial electron transport chain [9,12]. In addition, an imbalance in the cellular redox state results in cellular damage via lipid peroxidation, inflammation, mitochondrial damage, and apoptosis in various organs [9]. PQ poisoning can result in ulcers on the skin, lips, tongue, pharynx, and esophagus. Additionally, systemic effects can lead to multi-organ failure, including liver damage, acute kidney injury, respiratory failure, and convulsions [9,13,14,15]. The quantitative analysis of PQ levels in human samples helps to determine the cause of death, while autopsy and histopathology investigations provide additional support in elucidating the toxicokinetic and toxicity mechanisms involved [16]. Mortality rates of PQ poisoning reported by several studies were 33.0% to 91.7%, mainly from multiple organ failure and pulmonary fibrosis [17]. The high fatality rates resulting from PQ poisoning can be attributed to the limitation of effective treatments and specific antidotes [18,19]. Currently, there is no specific antidote or universally accepted treatment guidelines for PQ intoxication [9,13,14]. The available medical management options range from providing supportive care, including gastrointestinal tract decontamination within the first two to four h of ingestion, to utilizing various combinations of immunosuppressive therapies (dexamethasone and cyclophosphamide), antioxidants (vitamin C, vitamin E, and N-acetyl cysteine), and hemoperfusion within the initial two h of ingestion [9,13].

In addition to acute intoxication from PQ exposure, which can be fatal, long-term exposure has been associated with serious health conditions such as Parkinson’s disease (PD) [20,21,22]. Epidemiological investigations have explored the association between ambient paraquat dichloride exposure and PD risk in agricultural regions, involving a cohort of 829 PD patients and 824 controls. This study evaluated proximity to PQ applications in both residential and workplace settings. Findings indicate that individuals exposed to elevated levels of PQ, both at home and at work, exhibit an increased risk of developing PD. Specifically, working near PQ applications annually [OR = 2.15, 95% CI = 1.46–3.19] and experiencing higher exposure intensity [per 10 pounds (4.54 kg), OR = 2.08, 95% CI = 1.31–3.38] are strongly correlated with an increased risk of PD. Similar associations were observed for residential exposure [21]. A systematic review and meta-analysis further confirmed this association. Out of 7309 articles reviewed, 13 case-control studies encompassing 3231 PD patients and 4901 controls, along with one prospective cohort study, were included in the analysis. The meta-analysis demonstrated an overall odds ratio of 1.64 (95% CI = 1.27–2.13; I^2^ = 24.8%), suggesting a significant association between PQ exposure and PD [22]. Additionally, in vivo experimental studies have shown that PQ induces lesions in the substantia nigra, pars compacta, leading to neurotoxicity and dopaminergic degeneration. Despite these findings, evidence from human studies remains limited, underscoring the need for further research to fully elucidate the connection between PQ exposure and PD in human populations [20].

The quantitative analysis of PQ in human samples is crucial for forensic applications, as it helps to determine the cause of death [16]. Studies have shown that when the plasma PQ concentration is higher than 1.0 µg/mL within 24 h of ingestion, the probability of survival is less than 10% [18]. PQ is analyzed using various methods, including high-performance liquid chromatography-tandem mass spectrometry (HPLC-MS/MS), gas chromatography-mass spectrometry (GC-MS), ultraperformance liquid chromatography-tandem mass spectrometry (UPLC-MS/MS), and liquid chromatography-tandem mass spectrometry (LC-MS/MS), as summarized in Table 1 [16,23,24,25,26,27,28]. The method of choice for PQ analysis is LC-MS/MS with electrospray ionization (ESI) due to its sensitivity and selectivity, aligning with the water-soluble nature of PQ and its status as a doubly charged cationic species in solution [24,29]. The analytical separation achieved using the Hydrophilic Interaction Liquid Chromatography (HILIC) silica column demonstrated sensitivity and precision for highly polar compounds, eliminating the need for ion-pair reagents [24,29]. Additionally, this approach has been previously applied for analyzing PQ in urine and serum samples, making it a reliable tool for detecting PQ poisoning [24]. In this study, LC-MS/MS, which is a simple, sensitive, specific, and rapid screening analysis methodology, was used to analyze PQ [23,24].

In Thailand, few studies have been published on fatal PQ poisoning, including autopsy findings, histopathology, and quantitative analysis in post-mortem samples. One study reported acute PQ poisoning in seven autopsy cases. Blood PQ levels on admission were 0.04 to 4.27 µg/mL. The major causes of death were circulatory collapse, acute alveolar injury, acute tubular necrosis, hepatic necrosis, and cerebral edema. Histopathological examination revealed additional findings of pulmonary damage, bile duct injury, centrilobular cholestasis and necrosis, fatty metamorphosis, cortical necrosis, brain hemorrhage, and myocarditis [10]. Another case report of a Thai male farmer showed that dermal exposure to PQ solution led to serious systemic toxicity, such as renal failure, respiratory failure, and hepatic damage [30]. In support of these previous data, our study focused on the autopsy findings, histopathology, and quantitative analysis of PQ in post-mortem human serum samples using LC-MS/MS.

## 2. Materials and Methods

This study was a cross-sectional retrospective review carried out at the Department of Forensic Medicine in a tertiary care teaching hospital in Thailand from 1 January 2016 to 31 December 2019. Autopsies were performed on all deaths due to PQ poisoning, and the relevant viscera, serum, and urine were sent to the toxicology laboratory for chemical analysis. Toxicological analysis was performed using urine sodium dithionite test and LC-MS/MS. No specific sample size was calculated, and all confirmed cases of PQ poisoning were included in the study. Data for this study was collected from the post-mortem records of all confirmed cases.

### 2.1. Ethical Approval

The study was conducted following the Declaration of Helsinki and approved by the Institutional Review Board (Human Ethics Committee), the Faculty of Medicine, Chiang Mai University, Thailand. The ethics approval reference number is No. EXEMPTION 9375/2023. The victim’s family consent was not required because this study was retrospective and reviewed the pre-existing confidential database from the hospital. The results of this study were reported anonymously.

### 2.2. Case Reports and Autopsies

This study included seven fatal cases of PQ poisoning, comprising five men and two women. PQ intoxication was screened using the urine sodium dithionite test and subsequently confirmed by measuring serum PQ concentration through LC-MS/MS. Autopsies were performed, and samples of cardiac blood, femoral blood, urine, brain, heart, lung, liver, spleen, kidney, adrenal gland, esophagus, stomach, and intestine were collected. Serum samples were directly subjected to LC-MS/MS procedures, while the samples of brain, heart, lung, liver, spleen, kidney, adrenal gland, esophagus, stomach, and intestine were subjected to qualitative histopathological analysis using hematoxylin and eosin (H&E) staining (Sigma-Aldrich, St. Louis, MO, USA). 

The histopathological examination was conducted at the Department of Pathology, Chiang Mai University. Tissue samples were initially fixed in a 10% buffered formaldehyde solution. Following fixation, the samples underwent routine histopathological processing, which involved paraffin embedding, sectioning, and staining with H&E. This standard procedure enabled the clear visualization of cellular and tissue architecture under light microscopy. The histological slides were reviewed by both a histopathologist and a forensic doctor to confirm the cause of death. Consequently, the evaluation criteria focused on identifying specific pathological changes in cellular or tissue structures that could assist in determining the cause of death [31].

**Table 1:** Reported fatal cases and analytical methods of paraquat poisoning.

Paraquat Concentration (µg/mL)				Number of Cases	Method of Detection	Reference
Blood	Urine	Gastric Content	Other Samples			
0.5–372.0	N/A	N/A	N/A	7	LC-MS/MS	Present study
0.3–2.2 (Heart) 1.0–1.6 (Peripheral)	0.1–5.3	0.6–1.7	0.2–6.1 (Bile) 0.2 (Vitreous humor)	4	HPLC-MS/MS	[27]
150.0 (Heart) 80.0 (Peripheral)	910.0	N/A	320.0 (Bile) 60.0 (Vitreous humor)	1	HPLC-MS/MS	[28]
291.5 * (2.9–1108.8)	N/A	6028.3 * (3.0–21,617.2)	200.0 (Vitreous humor)	16	LC-MS/MS	[23]
3.3 * (0.1–9.5)	3.0 * (0.1–13.5)	N/A	4.1 * (0.5–11.9) (mg/g of lung)	5	GC-ion trap MS	[19]
68.7 * (1.5–335.9)	N/A	N/A	N/A	7	HPLC (Liquid-Liquid-Extraction)	[32]
5.1	6.0	17.2	80.6 (mg/kg of kidney)	1	HPLC-DAD	[33]
30.1–636.6	N/A	N/A	N/A	24	Spectrophotometry	[34]
2.0	5.1	N/A	52.1 (µg/g of kidney)	1	HPLC	[25]
7.9 * (0.2–25.0)	192.0	N/A	41.5 * (0.3–146.0) (µg/g of liver)	5	HPLC ion-pair extraction	[35]
7.2 * (1.1–13.3)	N/A	N/A	N/A	3	Spectrophotometry	[36]

* Mean, GC-ion trap MS, Gas chromatography-ion trap mass spectrometry; HPLC-DAD, high-performance liquid chromatography with diode-array detector, N/A represents “not available”.

### 2.3. Toxicological Evaluation 

#### 2.3.1. Reagents 

Paraquat dichloride hydrate (PQ) was purchased from Dr. Ehrenstorfer GmbH (Augsburg, Germany). Ethyl viologen dibromide (EV) was purchased from Sigma-Aldrich (St. Louis, MO, USA). Acetonitrile was purchased from J.T. Baker (Phillipsburg, NJ, USA). Ammonium formate was purchased from LOBA CHEMIE PYT. LTD. (Mumbai, Maharashtra, India). Formic acid was purchased from Fisher Scientific (Hampton, NH, USA). All chemicals were mass spectroscopy grade. 

#### 2.3.2. Preparation of Standards and Quality Control (QC) Samples

The stock solutions of PQ and EV were prepared at a concentration of 1 mg/mL in deionized water (DW). To prepare a 100 µg/mL PQ working solution, 10 µL of the stock solution was diluted with 100 µL of water. Six PQ calibration standards were then created by adding the working solutions to blank human serum, covering a range from 50 to 2000 ng/mL (50, 100, 200, 500, 1000, and 2000). QC samples at three concentration levels (75, 600, and 1500 ng/mL) were also prepared using the same procedure. The stock and working solutions were stored at 4 °C.

#### 2.3.3. Forensic Sample Preparation 

For each case, a serum sample of 250 µL was mixed with 500 µL of cold acetonitrile (−20 to −10 °C) and 20 µL of the internal standard (EV, 5 µg/mL). Next, the mixture underwent vortexing (Vortex-Genie 2 mixer, Scientific Industries, Inc., New York, NY, USA) at a speed of 2700 rpm for 10 s, followed by centrifugation (Centrifuge 5810 R, Eppendorf, Hamburg, Germany) at 14,500 rpm for 15 min at 4 °C. Finally, 10 µL of the supernatant was introduced into the LC-MS/MS system [24].

#### 2.3.4. Method Validation

##### Linearity and Lower Limit of Quantification (LLOQ)

Linearity was determined by evaluating PQ calibration standards covering a range from 50 to 2000 ng/mL. Six PQ calibration standards were generated by introducing working solutions into a blank human serum. To establish the calibration curve, a weighted linear least-squares regression (with a weight of 1/x) was employed, correlating the peak area ratios of PQ to EV with their corresponding concentrations. Regarding the Lack of Fit statistic, if the *p*-value is greater than the significance level (0.05), it suggests that the Lack of Fit is not statistically significant and the model fits well. The LLOQ was established as the lowest concentration on the calibration curve, determined through visual evaluation, at which the analyte could be reliably detected with acceptable precision and accuracy. The observed value was anticipated to fall within ±20% of the expected value [24,37]. Determining the LLOQ involved utilizing a blank serum sample and signal-to-noise ratios of 3 [24,37].

##### Accuracy and Precision

The QC samples were analyzed at three concentration levels (75, 600, and 1500 ng/mL) within the same day to assess intra-day accuracy and precision or on consecutive days to study inter-day accuracy and precision. Intra-day accuracy and precision were assessed by analyzing five replicates of QC samples at three concentration levels within a single run. To evaluate inter-day accuracy and precision, three replicates of QC samples were analyzed on four different validation days. Precision was indicated by the coefficient of variation (% CV), with the target result within ±15% for accuracy and reliability [24,38]. Accuracy was expressed as (mean concentration)/(spiked concentration) × 100%.

#### 2.3.5. Procedure of Analysis and LC-MS/MS Instrumentation 

PQ in post-mortem human serum samples was assayed by modifying a previously described procedure using LC-MS/MS [24]. The LC-MS/MS system consisted of an Agilent 1290 Infinity HPLC system coupled with a 6460 triple quadrupole mass spectrometer (Agilent Technologies, Inc., Santa Clara, CA, USA). Quantitative data analysis was performed and processed using the MassHunter software (version B.04.01, Agilent Technologies). Chromatographic separation of PQ was performed on a Poroshell 120 HILIC-Z column (100 × 2.1 mm I.D., 2.7 µm particle size, Agilent Technologies, Inc., Santa Clara, CA, USA). The mobile phase was a mixture of solvent A (20 mM ammonium formate containing 0.8% formic acid in DW at pH = 2–3) and solvent B (20 mM ammonium formate containing 0.8% formic acid in 10% acetonitrile at pH = 3–4), with a solvent ratio of 20:80 *v*/*v*. This mixture was delivered using isocratic elution. The column temperature was set at 30 °C, and the flow rate was 0.3 mL/min for a total runtime of six min. Tandem mass spectrometry was used to detect PQ and EV (used as an internal standard) using ESI in positive ion mode. The precursor ions were fragmented by varying the fragment voltage to obtain product ions. The collision energy for PQ and EV is presented in Table 2. Multiple reaction monitoring (MRM) technique was used for the selective quantification of PQ. Additionally, the second *m*/*z* to first *m*/*z* ratio must be consistent with the calibrators and within a tolerance of ±20% to confirm the presence of PQ in the samples [24].

## 3. Results

### 3.1. Demographic Characteristics, Autoptic, and Macroscopic Findings

**Figure 1:**
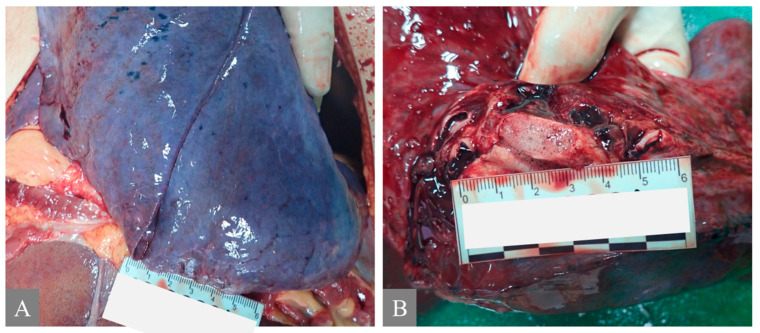
Autoptic macroscopic pathomorphological findings of the lung. (**A**) shows pulmonary congestion from Case 7. (**B**) shows pulmonary edema: frothy fluid in the main bronchi from Case 7.

In this study, seven fatal cases of PQ poisoning were included, comprising five men and two women aged between 39 and 70. The most prominent autoptic and macroscopic findings were observed in the lungs, which exhibited an increased weight due to edema, congestion, hemorrhage, and fibrosis (Figure 1). The liver and kidneys were also significantly altered, with liver steatosis, liver jaundice, and acute kidney injury being present. Additionally, erosion ulcers, corrosive burns, mucosal damage, and gastritis were observed due to the potential irritation caused by PQ. This study also found alterations in the heart, which included petechial hemorrhage, myocardial hemorrhage, and coronary occlusion. The exact amount of PQ ingested was unknown, and four out of the seven cases resulted in immediate death. The remaining three cases were admitted to the hospital in a serious condition but unfortunately died. In Case 6, the amount of ingestion was based on the patient’s past medical history. A summary of the autoptic and macroscopic findings is presented in Table 3 and Table 4.

### 3.2. Microscopic Histopathological Findings

**Figure 2:**
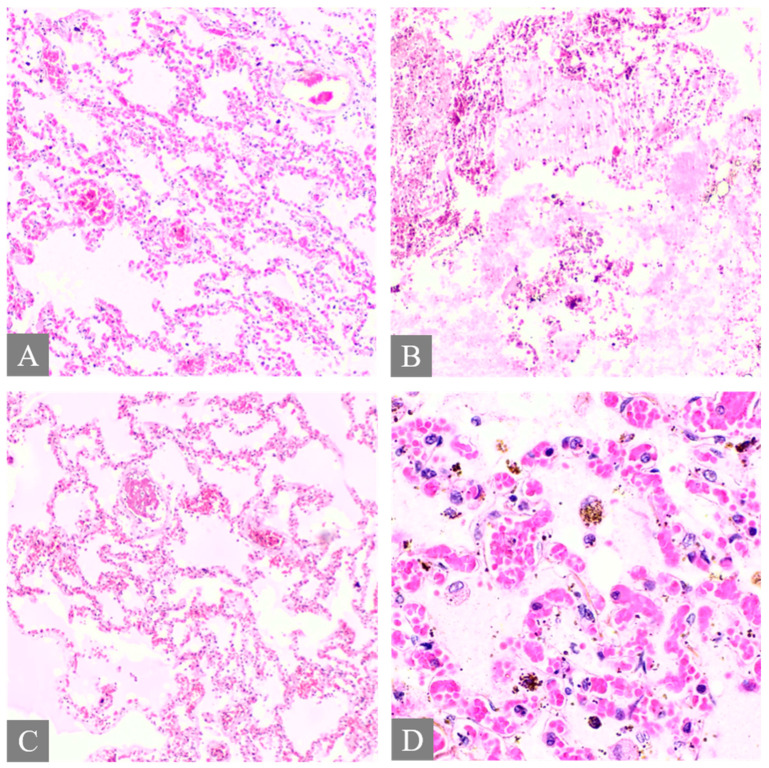
Histopathology of the lung. (**A**) shows pulmonary congestion from Case 7 (H&E, 10×). (**B**) shows diffuse alveolar damage from Case 3 (H&E, 10×). (**C**) shows fibrin platelet thrombi from Case 3 (H&E, 10×). (**D**) shows pulmonary hemosiderin indicating hemorrhage from Case 3 (H&E, 40×).

**Figure 3:**
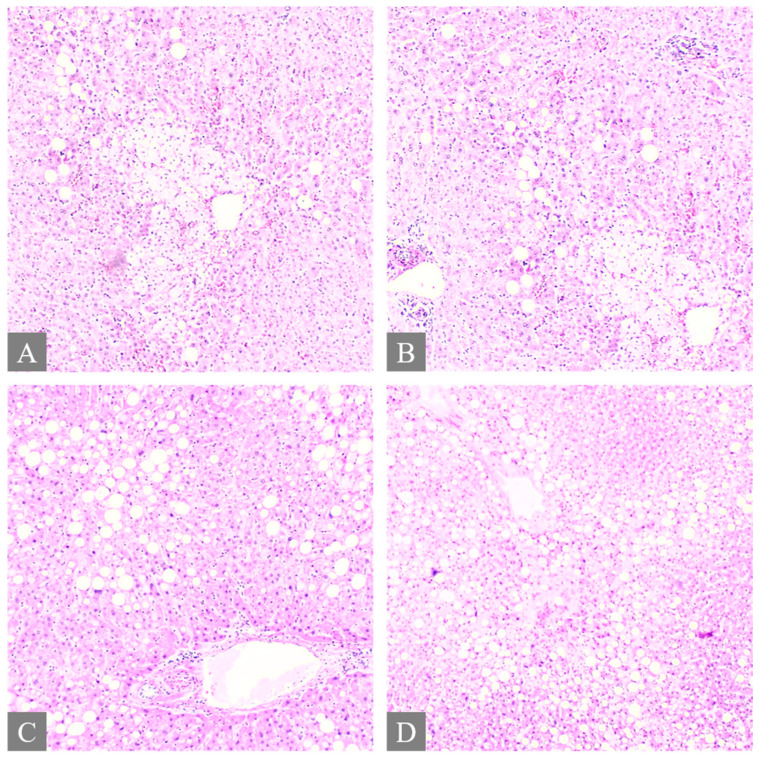
Histopathology of the liver. (**A**,**B**) shows multifocal ballooning degeneration of hepatocytes and liver steatosis from Case 3 (H&E, 10×). (**C**) shows liver steatosis from Case 7 (H&E, 10×). (**D**) shows liver steatosis from Case 7 (H&E, 5×).

**Figure 4:**
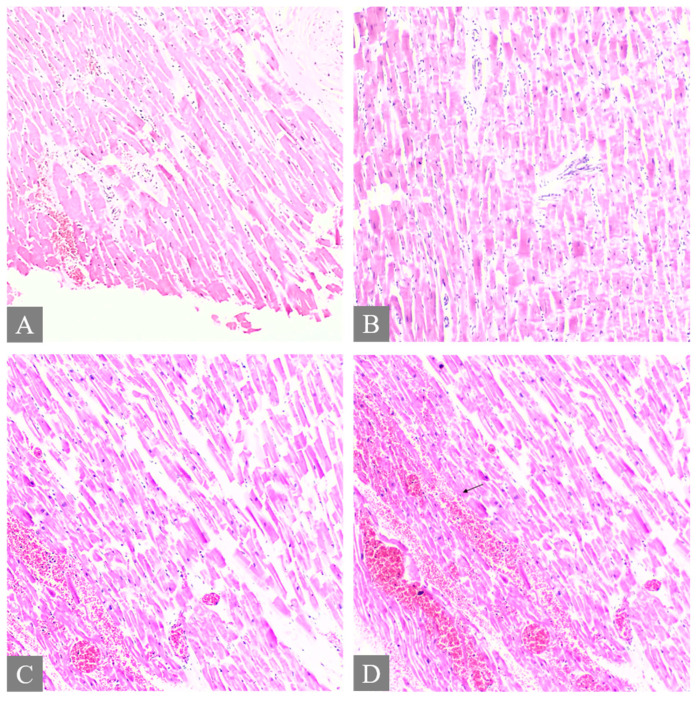
Histopathology of the heart. (**A**,**B**) shows myocardial necrosis with contraction band lesion, thrombosis, and focal subendocardial hemorrhage from Case 3 (H&E, 10×). (**C**) shows hemorrhage in the myocardium from Case 7 (H&E, 10×). (**D**) The arrow shows hemorrhage in the myocardium from Case 7 (H&E, 10×).

**Figure 5:**
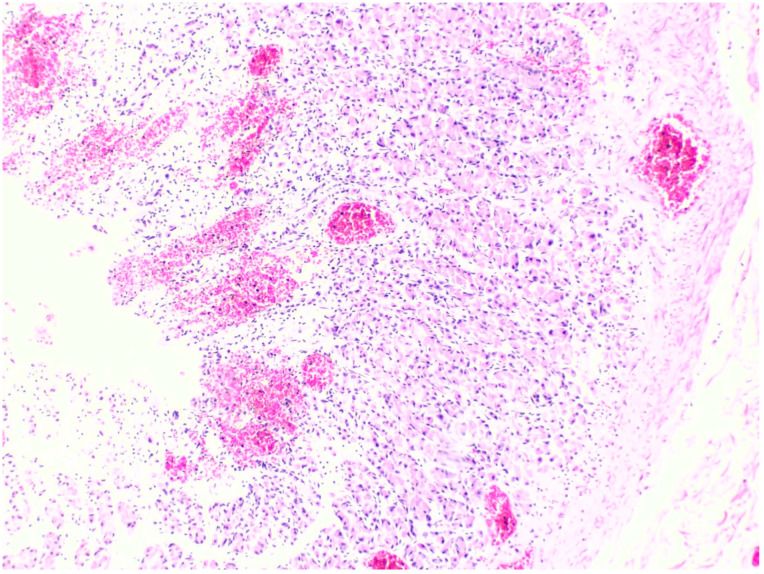
Histopathology of the stomach shows superficial mucosal hemorrhage from Case 7 (H&E, 10×).

**Figure 6:**
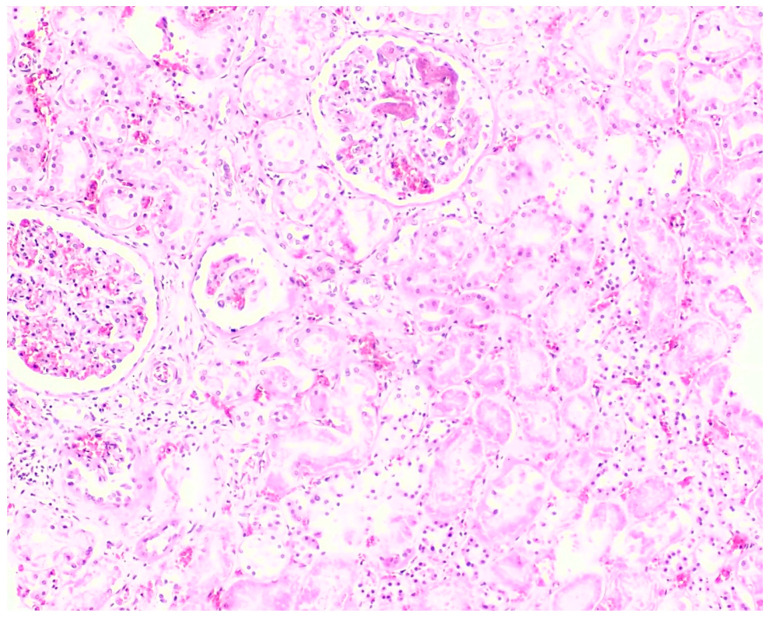
Histopathology of the kidney shows acute tubular necrosis and the presence of fibrin platelet thrombi in glomeruli from Case 3 (H&E, 10×).

**Figure 7:**
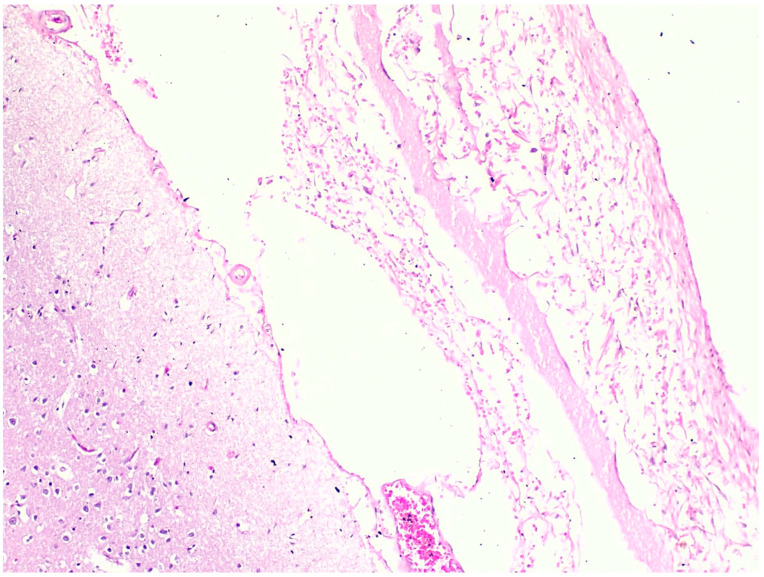
Histopathology of the brain shows red blood cells within the subarachnoid space from Case 7 (H&E, 10×).

All fatal cases exhibited pulmonary histological alterations, except for Case 2, for which lung histological data were not available due to post-mortem changes. The most notable histological findings were edema and congestion, accompanied by diffuse alveolar damage, fibrosis, petechial hemorrhage, and the presence of fibrin-platelet thrombi (Figure 2). Additionally, hepatic histological changes were observed in six out of the seven fatal cases. The most prominent histological finding was steatosis, and hepatocyte degeneration and canaliculi cholestasis were also observed (Figure 3). Cardiac histopathology was present in five out of the seven fatal cases, which included hemorrhage at the subendocardial and myocardial levels, as well as thrombosis, atherosclerosis, coronary vessel occlusion, myocardial necrosis, and infarction (Figure 4). Lesions in the esophagus and stomach were also observed, likely due to irritation. The histological findings showed mucosal hemorrhage, infarction, and inflammation (Figure 5). Partial autolysis was observed in the kidneys, along with the adrenal gland. Renal histological alterations included acute tubular necrosis and the presence of fibrin platelet thrombi in glomeruli (Figure 6). The adrenal gland exhibited focal cortical necrosis and lipid depletion of cortical cells, indicating cellular stress. Spleen congestion was found in three out of the seven fatal cases. Lesions in the brain were characterized by subarachnoid congestion, the presence of red blood cells within the subarachnoid space due to hemorrhage, and eosinophilic neurons, indicating acute neuronal injury (Figure 7). A summary of the microscopic histopathological findings is presented in Table 5.

### 3.3. PQ Quantification 

#### 3.3.1. Selectivity and Specificity

**Figure 8:**
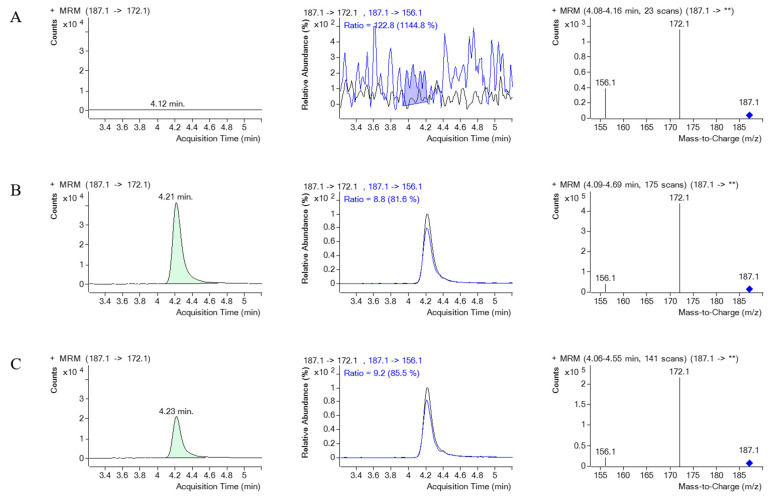
The MRM chromatograms of PQ, MRM of PQ and its product ion, along with a mass spectrum of PQ and its product ion spectrum in human serum. (**A**) represents blank serum, (**B**) represents blank serum spiked with PQ 1000 ng/mL, and (**C**) represents a real serum sample obtained from PQ poisoning cases. ** indicates the m/z of the precursor ion, which for PQ is 187.1, and then it is fragmented.

**Figure 9:**
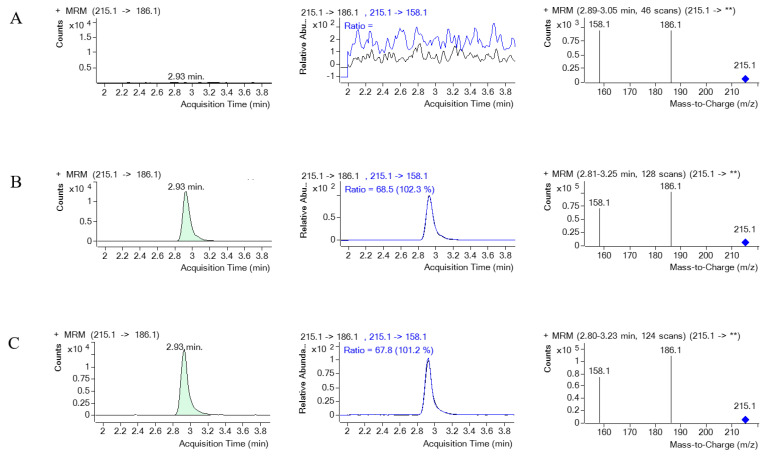
The MRM chromatograms of EV (internal standard), MRM of EV and its product ion, along with a mass spectrum of EV and its product ion spectrum in human serum. (**A**) represents blank serum, (**B**) represents blank serum spiked with EV, and (**C**) represents a real serum sample obtained from PQ poisoning cases. ** indicates the m/z of the precursor ion, which for EV is 215.1, and then it is fragmented.

Figure 8 and Figure 9 illustrate the MRM chromatograms of the standard and internal standard observed in serum, respectively. Notably, there was no occurrence of an endogenous interference peak at the retention time (t_R_) of PQ (t_R_ = 4.2 min) or EV (t_R_ = 2.9 min), which indicates that the method employed in this study has reasonable selectivity and specificity.

#### 3.3.2. Linearity of Calibration and LLOQ

The plotted calibration curves of PQ demonstrated linearity within the range of 50 to 2000 ng/mL, yielding an R^2^ value of 0.9997, with the regression equation y = 1.6006x − 0.0276. Regarding the Lack of Fit statistic, the *p*-value being higher than 0.05, indicating the absence of a significant Lack of Fit, implies that the linear model was an appropriate fit for the selected calibration ranges. The method’s LLOQ was determined to be 50.0 ng/mL.

#### 3.3.3. Accuracy and Precision

The intra- and inter-day precision in serum were within the 10% range, and the accuracy of PQ determination in plasma was between 101.2 and 110.9%. A summary of the intraday and inter-day precision and accuracy is presented in Table 6.

### 3.4. Application

The successful application of the developed method involved analyzing seven human serum samples. The retention time and precursor and product ion spectra of PQ in the samples were consistent with those of the genuine standard, indicating a good match.

PQ concentrations in serum (µg/mL) collected post-mortem were presented in Table 7 as independent levels for each reported case due to the unknown exact amount ingested and the time after ingestion. No data are available regarding the survival period, medical interventions (especially gastrointestinal decontamination), vomit volume, or the interval between exposure and serum sample collection. Therefore, no average concentrations could be calculated. This study reports fatal serum PQ levels ranging from 0.5 to 372.0 µg/mL.

## 4. Discussion

Acute PQ poisoning is an important health concern worldwide because of the high mortality rate [1]. In support of the limited published data, our study reported the autopsy findings, histopathology, and quantitative analysis of PQ in fatal cases. Pulmonary lesions were found in all cases, which were edema, congestion, hemorrhage, and fibrosis. Moreover, diffuse alveolar damage, petechial hemorrhage, and the presence of fibrin platelet thrombi were also presented. The lung is the important target organ for PQ toxicity [39,40]. PQ can be rapidly absorbed through ingestion and selectively accumulate in the lungs via an energy-dependent process involving an amino acid pump [9,41]. Previous post-mortem studies have reported that the highest concentrations of PQ were detected in lung samples, and pulmonary changes were found in all fatal cases [19,42]. The most prominent pulmonary toxic effect is the development of edema and diffuse alveolar damage, which presents 24 to 48 h after ingestion and is associated with extensive injury [39,42]. PQ generates oxygen-free radicals that damage the endothelial cells of alveolar capillaries and pneumocytes [43]. Previous post-mortem studies have reported major pathology, including fibrin deposition, alveolar-capillary endothelium damage, and hemorrhage. Diffuse alveolar damage was observed in the early exudative phase, followed by fibroblast proliferation and collagen deposition in the late proliferative phase [43]. In addition, in vivo studies have found endothelial damage leading to capillary congestion, which is proposed to be the cause of pulmonary edema [44]. 

PQ has a large volume of distribution (1.2 to 1.6 L/kg) and is distributed into all organs, especially the kidneys and liver. Additionally, PQ metabolism is limited, and over 90% of it is excreted unchanged in urine through tubular filtration and active tubular secretion [9,19,41]. Therefore, the kidneys could also be a target organ for PQ toxicity. This study found renal alterations, including acute tubular necrosis and the presence of fibrin platelet thrombi in glomeruli. Previous post-mortem studies have reported high PQ concentrations detected in all renal samples, supporting that the kidneys are a significant route of PQ elimination. These studies also observed interstitial hemorrhage, collagen deposition, necrosis of the proximal tubules, and glomeruli, which are consistent with our findings [19,45]. Moreover, a clinical study reported an increased incidence of acute kidney injury in patients with acute PQ poisoning [46]. Regarding the toxicokinetics of PQ, the liver could also be another target organ. Our findings showed hepatic changes, including steatosis, hepatocyte degeneration, and canaliculi cholestasis. Previous post-mortem studies have also reported consistent results, including hepatic damage, centrilobular cholestasis, and cholangiocellular injury [19,47]. This study suggested that PQ-induced liver damage in humans occurs in two phases. The initial phase, occurring in the first 48 h, causes hepatocellular damage due to the accumulation of the parent compound. The second phase results in cholangiocellular and cholestatic damage, which may be related to the excretion of unmetabolized PQ or a metabolite into bile [19,47]. Damage to the bile secretory apparatus in hepatocytes suggested that PQ might target both the bile secretory apparatus and biliary epithelial cells, and PQ excretion could occur through the biliary route facilitated by P-glycoprotein. The presence of PQ in post-mortem bile samples supports the idea of enterohepatic recirculation in humans [19,47,48,49].

Interestingly, cardiac histopathology was present, which included hemorrhage, as well as thrombosis, coronary occlusion, myocardial necrosis, and infarction. An in vivo study showed that ingesting a high dose of PQ resulted in death within hours, with a direct and strong relationship between PQ concentration in the heart and survival time. The study suggests that the rapid accumulation of PQ in the heart may play a significant role in acute death [50]. Lesions in the brain, including subarachnoid congestion, hemorrhage, and acute neuronal injury, were also reported in this study. In vivo studies showed that the prefrontal cortex and hypothalamus had the highest levels of PQ, and the concentrations in the prefrontal cortex may have contributed to neuronal cell death in rats [51]. This study reported that there were signs of cellular stress in the adrenal gland, as evidenced by focal cortical necrosis and lipid depletion of cortical cells. The in vivo study examined the effects of a single sub-lethal dose of PQ on the adrenal glands. Histological examination revealed congestion in the adrenal cortical sinusoids and blood vessels of the medulla, potentially due to the formation of peroxynitrite anion and its role in PQ toxicity [52].

The impact of PQ restrictions on death rates is significant and requires long-term monitoring. Our research, conducted from 2016 to 2019, predates the PQ ban implemented on 1 June 2020 [53]. Currently, there is limited evidence regarding the effect of the PQ ban on death rates from PQ intoxication in Thailand. A study by the Ramathibodi Poison Center, a prominent institution in Thailand, reported no significant change in occupational PQ exposure cases before and after the ban, suggesting that residual quantities of PQ may have remained in the country [54]. In contrast, studies from Korea, Taiwan, and Sri Lanka have shown a reduction in suicide rates related to pesticide use, including PQ, following its prohibition [55,56,57,58,59,60,61]. This finding is consistent with our unpublished data from the Department of Forensic Medicine, Faculty of Medicine, Chiang Mai University, which indicates that no cases of PQ intoxication were found after the ban. However, as these data are limited to a single center, it may not fully reflect the broader impact of the ban. Additionally, our study shows that PQ poisoning cases are extremely rare, with only seven fatal cases identified between 2016 and 2019. Regarding the study, in the hospital setting in Thailand, the majority of PQ poisoning cases were managed in hospitals, with fatalities often occurring later due to complications, such as lung fibrosis and multi-organ failure, rather than immediate death or requiring forensic investigation [15]. This underscores the need for comprehensive nationwide monitoring. Future research should focus on evaluating the broader impact of the PQ restriction across various settings to better understand its effectiveness in reducing PQ-related fatalities. Comprehensive and extensive data collection is essential for assessing the long-term impact of the PQ ban.

The implications of PQ poisoning are multifaceted, encompassing health, safety, ethical concerns, and corporate responsibility. Victims and their families experience significant health and psychological impacts, with ethical issues arising when prompt and appropriate medical care is lacking [62,63]. The role of regulatory agencies in permitting PQ use is questioned, particularly in countries lacking adequate safety measures [62]. Social implications include public awareness and education about PQ risks. Legally, the Thai government has implemented several policies to reduce pesticide use and associated risks. However, the current enforcement of these policies remains incomplete [63]. One successful outcome of the enforcement is that PQ was banned in Thailand on 1 June 2020 [53]. Unfortunately, a study by the Ramathibodi Poison Center reported no significant change in occupational PQ exposure cases before and after the ban, suggesting that residual quantities of PQ may have remained in the country [54]. Promoting consumer education regarding the safe use of agricultural products and raising awareness of their toxicity is crucial for minimizing the risk of misuse. Additionally, implementing strategies aimed at sustainably reducing pesticide usage is imperative to mitigate potential health hazards. Several studies have reported that the prohibition of PQ correlates with a decrease in suicide rates [55,56,57,58,59,60,61]. Thus, PQ poisoning could be prevented through collaborative efforts involving the government, the agricultural sector, the healthcare domain, environmental advocates, and private industries [63].

The future directions for research and practice on PQ poisoning in the forensic science field are important for contributing to accurate and effective investigations, particularly in cases of suspected poisoning. Studying toxicokinetics in humans is of interest, especially post-mortem distribution/redistribution, to identify target organs and discover non-invasive biomarkers. It is essential to develop sensitive methods for detecting PQ in biological samples, considering stability and advanced techniques. Additionally, establishing the criteria to classify severity using histopathology and employing toxicokinetics to predict the timing and amount of exposure are crucial aspects of this endeavor.

## 5. Conclusions

In conclusion, this study provides comprehensive data on fatal PQ poisoning, including autopsy findings, histopathology, and quantitative analysis in post-mortem samples. The data can be valuable in establishing definitive diagnoses in post-mortem cases and potentially guiding appropriate treatment strategies based on the mechanism of PQ toxicity. This value is important for clinicians and forensic investigators who are involved in the diagnosis and management of PQ poisoning cases. Future research and practices concerning PQ poisoning in forensic science are important for contributing to accurate and effective investigations. The findings of this study emphasize the importance of a multidisciplinary approach in investigating and diagnosing cases of PQ poisoning. Overall, this study provides valuable insights into the diagnosis, as well as the management of fatal PQ poisoning, and serves as a useful guide for future investigations into cases of PQ poisoning.

## Figures and Tables

**Table 2:** Precursor ions, fragment voltage, product ions, and collision energy for paraquat and ethyl viologen.

Chemicals	Precursor Ions (*m*/*z*)	Fragment Voltage (V)	Product Ions (*m*/*z*)	Collision Energy (V)
Ethyl viologen	215.1	92	186.1	14
			158.1	34
Paraquat	187.1	92	172.1	18
			156.1	46

**Table 3:** Demographic characteristics of fatal cases and autoptic findings.

Autopsy Case No.	Age	Gender	Weight (g)						Manner/Amount of Ingestion (mL)	Survival Time (Day)
			Brain	Heart	R/L Lung	Liver	Spleen	R/L Kidney		
1	44	M	N/A	310 (~)	740 (↑)/625 (↑)	1515 (~)	50 (~)	100 (~)/110 (~)	Suicide/N/A	N/A
2	45	M	1450 (↑)	344 (~)	710 (↑)/670 (↑)	1560 (↑)	105 (~)	145 (↑)/135 (↑)	N/A	N/A
3	39	M	1410 (~)	535 (↑)	1044 (↑)/1000 (↑)	N/A	N/A	N/A	Suicide/N/A	1
4	44	F	1381 (↑)	246 (~)	1009 (↑)/728 (↑)	1520 (↑)	88 (~)	141 (↑)/146 (↑)	N/A	2
5	70	F	1120 (↓)	295 (~)	779 (↑)/553 (↑)	1027 (↓)	120 (~)	140 (~)/115 (~)	N/A	N/A
6	54	M	978 (~)	253 (~)	270 (~)/322 (~)	1140 (~)	87 (~)	81 (~)/73 (~)	N/A/500	1
7	42	M	1356 (~)	380 (↑)	880 (↑)/773 (↑)	1683 (↑)	177 (↑)	210 (↑)/225 (↑)	N/A	N/A

Note: for each organ weight: (~), within normal range; (↑) increased weight; (↓) decreased weight.

**Table 4:** Macroscopic pathomorphological findings.

Autopsy Case No.	Heart	Lung	Liver	Stomach/Intestine	Kidney
1	Subendocardial, myocardial hemorrhage	Pulmonary edema, hemorrhage	Steatosis/necrosis, jaundice	Greenish blue color (lips, nose, larynx, trachea, stomach, intestine)	Acute kidney injury
2	N/A	Pulmonary edema, greenish blue color (trachea)	N/A	Greenish blue color (stomach, intestine)	N/A
3	Petechial hemorrhage, myocardial hemorrhage	Pulmonary congestion, hemorrhage	Steatosis	Gastritis	N/A
4	N/A	Severe pulmonary edema, intrapulmonary hemorrhage	Steatosis	Gastritis, mucosal hemorrhage, corrosive burn of esophagus	N/A
5	Congestion	Pulmonary fibrosis, edema, congestion	Jaundice	Mucosal damage	N/A
6	Petechial hemorrhage, coronary occlusion	Adhesion of all lobes, pulmonary hemorrhage	N/A	Erosion ulcer, petechial hemorrhage (esophagus, stomach), greenish-blue color (gastric content)	Acute kidney injury
7	N/A	Pulmonary edema, congestion	N/A	Gastritis	N/A

**Table 5:** Microscopic histopathological findings.

Autopsy Case No.	Brain	Heart	Lung	Liver	Spleen	Kidney	Adrenal Gland	Esophagus/ Stomach
1	N/A	Hemorrhage at subendocardial and myocardium	Edema	Steatosis	N/A	Partial autolysis	Partial autolysis	N/A
2	N/A	N/A	N/A	Steatosis	N/A	N/A	N/A	N/A
3	Subarachnoid congestion, hemorrhage, eosinophilic neurons	Thrombosis, myocardial necrosis, focal subendocardial hemorrhage, atherosclerosis	Diffuse alveolar damage, fibrin platelet thrombi	Multifocal ballooning degeneration (hepatocyte), steatosis	Congestion	Acute tubular necrosis, fibrin platelet thrombi in glomeruli	Focal cortical necrosis	Mucosal infarct, hemorrhage
4	N/A	N/A	Edema, congestion, petechial hemorrhage	Steatosis	Congestion	N/A	N/A	Mucosal hemorrhage, esophagitis
5	N/A	Atherosclerosis	Diffuse alveolar damage, hemorrhage, fibrosis	Cholestasis	N/A	Acute kidney injury	N/A	N/A
6	N/A	Myocardial infarction, coronary occlusion	Edema, congestion, petechial hemorrhage	N/A	N/A	N/A	Lipid depletion	Mucosal infarct, hemorrhage
7	Red blood cells within the subarachnoid space	Cardiomegaly, hemorrhage at myocardium	Edema, congestion,	Steatosis	Congestion	N/A	Lipid depletion of cortical cells	Superficial mucosal hemorrhage

**Table 6:** Intra- and inter-day precision and accuracy for paraquat in serum.

Nominal Concentration (ng/mL)	INTRA-DAY				Inter-Day			
	Mean Detected (ng/mL)	SD	Accuracy (%RR)	Precision (%CV)	Mean Detected (ng/mL)	SD	Accuracy (%RR)	Precision (%CV)
75.0	83.2	1.9	110.9	2.2	77.1	3.9	102.8	5.1
600.0	657.8	28.2	109.6	4.3	623.6	20.4	103.9	3.3
1500.0	1607.2	72.5	107.2	4.5	1517.7	104.9	101.2	6.9

**Table 7:** Paraquat concentration in serum (µg/mL) collected post-mortem.

Autopsy Case No.	Paraquat Concentration (µg/mL)
1	332.0
2	372.0
3	9.8
4	9.7
5	0.9
6	36.0
7	0.5

## Data Availability

All data and materials are included in the manuscript.
